# Point-of-Care Ultrasound in the Outpatient Management of Patients With Cirrhosis: Rapid and Accurate Decisions

**DOI:** 10.7759/cureus.85251

**Published:** 2025-06-02

**Authors:** Armando Antonio Baeza-Zapata, Luis C Chávez-García

**Affiliations:** 1 Department of Medicine, Hospital Angeles Chihuahua, Chihuahua, MEX; 2 Gastroenterology, Salvador Zubirán National Institute of Health Sciences and Nutrition, Ciudad de México, MEX

**Keywords:** decompensated cirrhosis, live cirrhosis, liver transplant, pocus (point of care ultrasound), ultrasound scan

## Abstract

The integration of point-of-care ultrasound (POCUS) into routine clinical practice has transformed traditional physical examination. Originally a radiologic tool reserved for imaging specialists, ultrasound is now a rapid, accessible modality that enhances bedside decision-making across multiple medical disciplines.

In patients with cirrhosis, POCUS offers a unique opportunity to detect early signs of decompensation. These findings, often undetectable by conventional examination, can lead to timely interventions that significantly impact prognosis, management, and transplant candidacy.

The role of bedside ultrasound in outpatient follow-up must be emphasized. Incorporating POCUS into routine evaluations can enhance clinical outcomes and support a paradigm shift toward more dynamic and responsive care for patients with cirrhosis.

## Editorial

This tool has evolved from a radiologic study performed exclusively by imaging specialists to an accessible and highly useful complement to physical examination. It enables timely clinical decision-making directly from the outpatient clinic [1]. Point-of-care ultrasound (POCUS) is now considered the “fifth pillar” of physical examination - alongside inspection, palpation, percussion, and auscultation. It can be performed in minutes and integrated seamlessly into routine clinical assessment, including during inpatient evaluations and outpatient follow-up [2].

Ultrasound has robust evidence supporting its use in trauma protocols within emergency departments. It has revolutionized pulmonary evaluation by improving the diagnosis of pleuropulmonary syndromes [3]. In critical care, POCUS is widely employed to assess volume status, shock etiology, response to fluid resuscitation, global cardiac function, and increasingly, to monitor neurocritical patients - particularly in evaluating and managing intracranial hypertension [4].

Patients with cirrhosis represent a vulnerable population requiring meticulous and continuous monitoring. The primary goal is to prevent or delay decompensation [5], which signals diminished hepatic reserve and often indicates the presence of an acute precipitating event - such as portal or mesenteric thrombosis, hepatocellular carcinoma (HCC), infection, or progression of the underlying liver disease [6]. The survival and quality of life of compensated patients differ drastically from those who become decompensated [7].

It is important to note that ultrasound-based detection of hepatic lesions for HCC surveillance, Doppler evaluation for portal or splenic vein thrombosis, and formal structural measurements should remain under the purview of radiologists, as these are beyond the intended scope of routine POCUS use [8].

Timely detection of signs of hepatic decompensation enables clinical intervention that may alter the patient’s follow-up, management strategy, and overall prognosis. When implemented correctly and systematically during routine outpatient visits, POCUS offers substantial clinical value. The identification of subtle changes can prompt further diagnostic studies to evaluate complications such as portal vein thrombosis or HCC, which may initially present only with ascites or signs of portal hypertension [9,10]. In such cases, for thrombosis, early initiation of anticoagulation therapy or locoregional/systemic treatment for HCC can help preserve candidacy for liver transplantation [11,12].

Conventional physical examination is markedly limited in detecting early decompensation. Even in expert hands, at least 1,000 mL of ascitic fluid is required for clinical detection, and pleural effusions must exceed 300 mL to become perceptible [13,14]. A greater challenge lies in identifying the transition from minimal to overt hepatic encephalopathy, which may present with a broad spectrum of neurological or psychiatric symptoms. Current diagnostic tools for this condition are labor-intensive and often subjective. In this context, objective assessments are crucial when patients present with subtle behavioral changes. For instance, the measurement of optic nerve sheath diameter (ONSD) is gaining traction as a practical, noninvasive method for early detection of cerebral involvement (Table 1) [6,15].

**Table 1:** Clinical Utility of Point-of-Care Ultrasound in Patients with Cirrhosis

Explored Region	Clinical Implication	Potential Intervention
Abdominal ultrasound	Evaluation of Morrison’s pouch, perihepatic, pelvic, and Koller spaces. Detection between 100 to 1000 mL of fluid	Sodium and fluid restriction. Search for new triggers of decompensation
Optic nerve sheath diameter (ONSD)	Detection of early hepatic encephalopathy. Ruling out causes of neurological deterioration that do not cause intracranial hypertension (delirium, withdrawal)	Consider noninvasive tests for encephalopathy. Search for new triggers of decompensation
Focused cardiac ultrasound (FoCUS)	Early detection of cirrhotic cardiomyopathy. Global cardiac function assessment	Adjustment of diuretic therapy; referral to cardiology if indicated
Inferior vena cava (IVC) index	Assessment of volume status and fluid responsiveness	Adjustment of diuretics in volume overload. Hospitalization for hypovolemia (dehydration, bleeding, infection)
Thoracic ultrasound	Early detection of pleural effusion (especially hepatic hydrothorax), identification of effusions <300 mL.	Sodium and fluid restriction. Unfavorable prognosis; Consider priority liver transplant evaluation in cases of hepatic hydrothorax

Conclusion

The routine integration of ultrasound into outpatient care for patients with cirrhosis provides a rapid, precise, and complementary physical examination tool. It offers objective data that supports timely clinical decisions and can significantly alter the disease trajectory. Early identification of ascites, pleural effusion, or encephalopathy may reflect declining hepatic reserve. Prompt management can address the underlying cause and preserve transplant eligibility when indicated. Moreover, cardiac function assessment, volume status evaluation, and identification of volume overload within seconds can help determine whether a patient with acute kidney injury may benefit from albumin administration or diuretics.

These simple, noninvasive interventions align with the primary goal of care in cirrhosis: to prevent or delay further decompensation. Any decline in hepatic reserve warrants etiologic investigation, appropriate imaging, and early referral for liver transplantation when necessary. Additionally, it is important to acknowledge the economic barriers that may limit the acquisition of ultrasound equipment and access to proper training, especially in resource-limited settings - particularly in countries where healthcare resources are scarce.
